# Adjustable Algorithmic Tool for Assessing the Effectiveness of Maternal Respiratory Syncytial Virus (RSV) Vaccination on Infant Mortality in Developing Countries

**DOI:** 10.1155/2021/5536633

**Published:** 2021-05-24

**Authors:** Rachel Cevigney, Christopher Leary, Bernard Gonik

**Affiliations:** ^1^Department of Obstetrics and Gynecology, Wayne State University School of Medicine, USA; ^2^SUNY Geneseo Department of Mathematics, USA

## Abstract

Acute lower respiratory infection (ALRI) due to RSV is a common cause of global infant mortality, with most cases occurring in developing countries. Using data aggregated from priority countries as designated by the United States Agency for International Development's (USAID) Maternal Child Health and Nutrition (MCHN) program, we created an adjustable algorithmic tool for visualizing the effectiveness of candidate maternal RSV vaccination on infant mortality. Country-specific estimates for disease burden and case fatality rates were computed based on established data. Country-specific RSV-ALRI incidence rates for infants 0-5 months were scaled based on the reported incidence rates for children 0-59 months. Using in-hospital mortality rates and predetermined “inflation factor,” we estimated the mortality of infants aged 0-5 months. Given implementation of a candidate maternal vaccination program, estimated reduction in infant RSV-ALRI incidence and mortality rates were calculated. User input is used to determine the coverage of the program and the efficacy of the vaccine. Using the generated algorithm, the overall reduction in infant mortality varied considerably depending on vaccine efficacy and distribution. Given a potential efficacy of 70% and a maternal distribution rate of 50% in every USAID MCHN priority country, annual RSV-ALRI-related infant mortality is estimated to be reduced by 14,862 cases. The absolute country-specific reduction is dependent on the number of live births; countries with the highest birth rates had the greatest impact on annual mortality reduction. The adjustable algorithm provides a standardized analytical tool in the evaluation of candidate maternal RSV vaccines. Ultimately, it can be used to guide public health initiatives, research funding, and policy implementation concerning the effectiveness of potential maternal RSV vaccination on reducing infant mortality.

## 1. Introduction

Globally, acute lower respiratory infection (ALRI) is a major contributor to infant morbidity and mortality. Respiratory syncytial virus (RSV) is one of the most commonly identified etiologic viral pathogens leading to ALRI [1–3]. According to Shi et al., an estimated 59,600 children less than 5 years old died in-hospital in 2015 from RSV-related ALRI. Infants less than six months of age made up 46% of the affected population. An overwhelming majority of cases of RSV-induced ALRI occur in developing countries [1].

The prevention and treatment of RSV poses a great financial burden; an estimated 50-70% of infants become infected with RSV during their first winter season [4]. Current treatment of RSV-related ALRI in children remains focused in supportive care, including respiratory support and hydration [3, 5, 6]. A humanized monoclonal antibody, Palivizumab, is the only available prophylactic treatment [7]. Due to the high cost, this medication is reserved for high-risk patients [7]. Additionally, natural infection has not provided long-term immunity or adequate amounts of neutralizing antibodies [8]. Reinfection rates can be as high as 83% depending on the season [9]. The high cost of care and lack of unanimously efficacious and widely available pharmaceutical interventions paired with and high incidence, reinfection, and death rates in infants <6 months old make potential maternal vaccination an attractive option in reducing disease burden [6].

An analysis of cost-effectiveness published by Cromer et al. compares the various strategies for preventing and treating RSV-related illness. The most economic strategies were single dose protection that could be given to specifically protect infants born just prior the start of RSV season [7]. Maternal vaccination during pregnancy, notably in the third trimester, can provide transient passive immunity to infants via immunoglobulin transfer through the placenta [10]. It is estimated that the antibodies can be protective for up to 6 months before decaying. Because a large portion of antibody transfer occurs in the third trimester, preterm infants are at a greater risk for severe infections that could be avoided with maternal vaccination [11].

Some ambiguity remains in identifying the immune response and target most effective in providing immunity to RSV [6]. The RSV F glycoprotein, a class I fusion protein, and the RSV F prefusion form have been major targets of interest for candidate vaccines. These proteins mediate viral entry and are essential to the pathogenicity of the virus [12]. A number of maternally indicated RSV F protein vaccines have been investigated both preclinically and through clinical trials. Novavax, a RSV F vaccine, has completed clinical trial and has been published [13]. Both Pfizer and GlaxoSmithKline have RSV prefusion F vaccines in phase 3 of clinical trial [14, 15].

**Figure 1:**
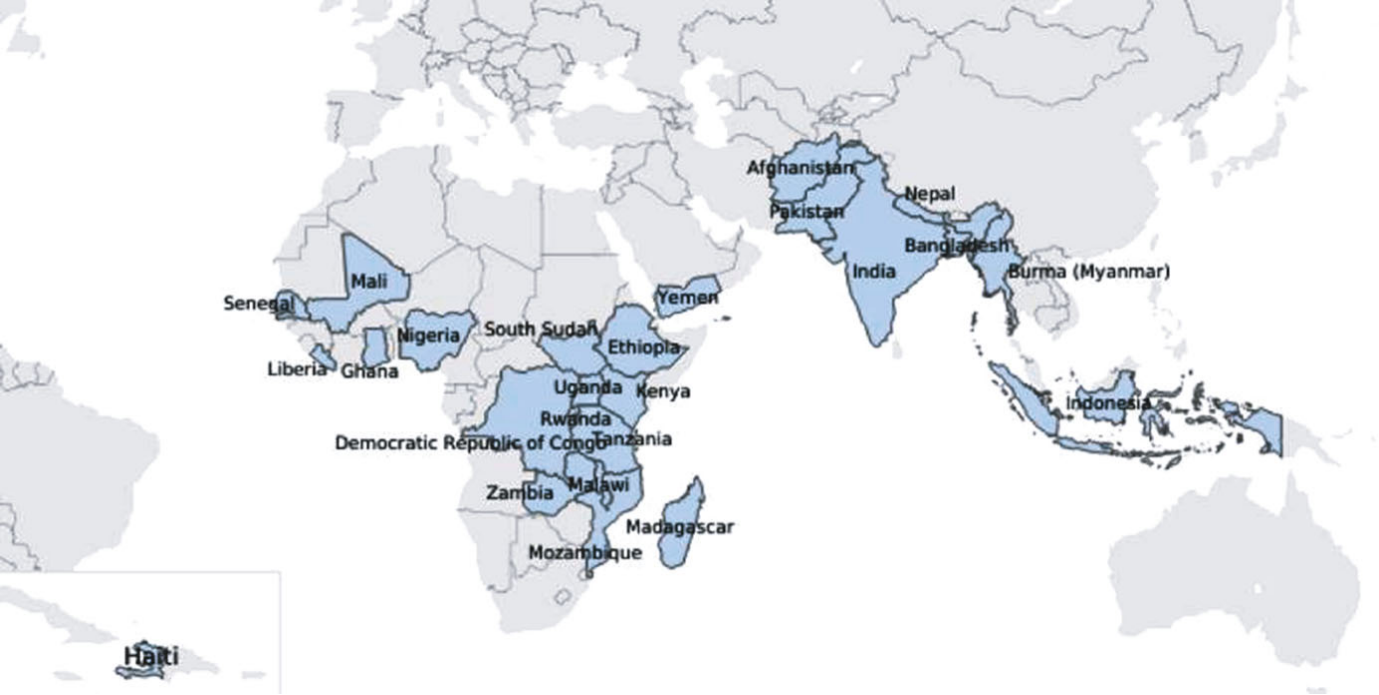
USAID's MCHN map. This map depicts USAID's MCHN priority countries that were investigated in creation of the algorithmic tool to assess effectiveness of candidate maternal RSV vaccination on reducing infant incidence and mortality in developing countries. Data for South Sudan were not available.

Though the need for an approved and effective RSV vaccination is currently unmet, we created an adjustable algorithmic tool for visualizing the effectiveness of candidate maternal RSV vaccination on reducing disease incidence and infant mortality. We used data specific to priority countries as designated by the United States Agency for International Development (USAID) Maternal Child Health and Nutrition (MCHN) program as depicted in Figure 1 [16]. This tool can be used to quantify the expected benefit of vaccination implementation.

## 2. Methods

Based on data from Shi et al. [1], USAID MCHN country-specific estimates for RSV disease burden and case fatality rates among infants age 0-5 months given implementation of a candidate vaccination program were computed. The population of interest is infants <6 months of age (0-5 months) because of the anticipated benefit of transient maternal antibody transfer. Data were collected, calculated, and reported by both country and region-specific estimates. Efficacy of the vaccine and distribution to pregnant women are determined by user input.

Absolute value of RSV incidence by country was calculated for infants age 0-5 months using the following scale. Regional RSV incidence rates were obtained from the literature for infants age 0-5 months (RI:0-5) and children age 0-59 months (RI:0-59). Using these rates as a scale along with the reported country-specific RSV incidence rates for children 0-59 months (CI:0-59), we estimated the country-specific RSV incidence rates for infants age 0-5 months (CI:0-5) by equating the regional and country-specific ratios using Equation (1).

(1) $$\begin{array}{c} \frac{\text{RI}:0-5}{\text{RI}:0-59}=\frac{\text{CI}:0-5}{\text{CI}:0-59}. \end{array}$$

**Figure 2:**
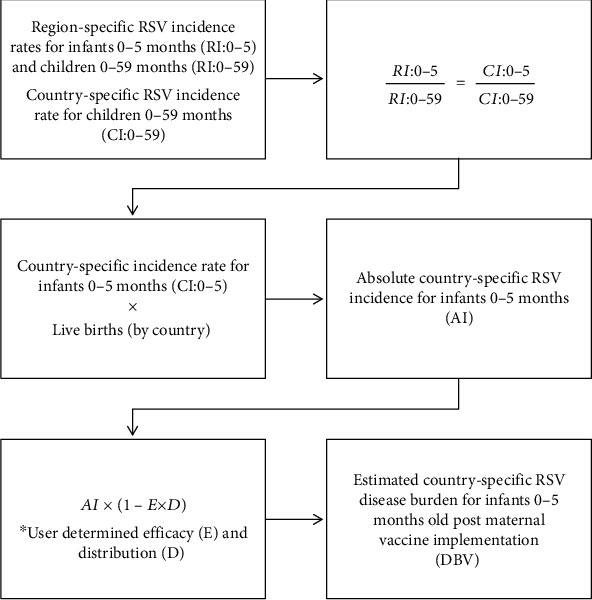
Flowchart of calculation of country-specific infant (0-5 months) RSV incidence.

Then, the country-specific incidence rate for infants 0-5 months old was multiplied by the number of live births in each country to obtain the absolute incidence of RSV for each country [10]. Using this value, and user determined values for efficacy and distribution of a candidate vaccine, the incidence of RSV given implementation of a candidate vaccination program is estimated using Equation (2). The process of the calculation of infant RSV incidence given candidate maternal RSV vaccination is displayed in Figure 2.

(2) $$\begin{array}{c} \text{AI}\times \left(1-E\times D\right)=\text{DBV}, \end{array}$$

AI:Absolute incidence

*E*:Efficacy

*D*:Distribution to pregnant women

DBV:Estimated RSV disease burden postmaternal vaccine implementation.

Infant RSV incidence was also calculated using region-wide incidence rate estimates. The regional RSV incidence rate for infants 0-5 months was multiplied by country-specific live birth rates yielding the absolute incidence of RSV in each country based on region wide estimates. Using this value, and user determined values for efficacy and distribution of a candidate vaccine, we estimate the reduction in incidence of RSV given implementation of a vaccination program.

Next, infant RSV mortality rate given implementation of a candidate maternal vaccination program was calculated. RSV hospitalization rates were reported by age bands: 0-1 month, 1-2 months, and 2-5 months. Using the country-specific number of live births and mortality rate, we first calculated the absolute number of infants age 0-1 month-old, 1-2 months old, and 2-5 months old [17]. Neonatal and infant mortality rates were obtained from http://data.unicef.org, using 2016 midyear estimates throughout [18, 19]. The mortality rates for infants 2-5 months old were calculated assuming that the mortality rate in each country was constant across months 2-12. The purpose of this calculation was to adjust the absolute number of infants in older age brackets and to account for infant mortalities that are unrelated to RSV.

**Figure 3:**
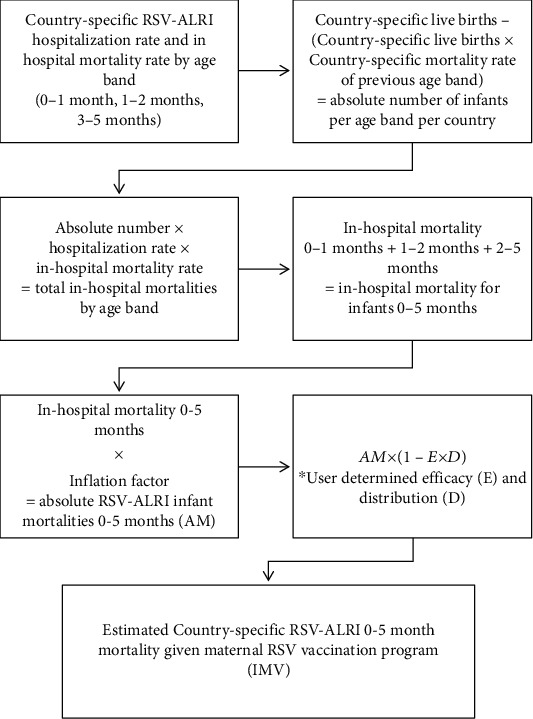
Flowchart of calculation of country-specific infant (0-5 months) RSV mortality.

We then multiplied the absolute number of infants in each age group by reported age-specific hospitalization rates and in-hospital mortality rates. Additionally, we multiplied by a predetermined inflation factor to account for infant RSV mortalities that occurred outside of the hospital setting [1]. The summation of the age bands results in the absolute RSV mortality for infants age 0-5 months. Using this value, and user determined values for efficacy and distribution of a candidate vaccine, we estimate the RSV infant mortality given implementation of a vaccination program using Equation (3). The process of the calculation of infant RSV mortality given candidate maternal RSV vaccination is displayed in Figure 3.

(3) $$\begin{array}{c} \text{AM}\times \left(1-E\times D\right)=\text{IMV}, \end{array}$$

AM:Absolute RSV-ALRI infant mortality

*E*:Efficacy

*D*:Distribution to pregnant women

IMV:Estimated RSV-ALRI infant mortality postmaternal vaccine implementation.

Infant RSV-ALRI mortality rate given implementation of a candidate maternal vaccination program was also calculated using region-wide estimates for hospitalization rates and in-hospital mortality rates.

We assumed that vaccination success was binary, in that the vaccine only prevents RSV and does not reduce virulence or severity of disease. Programing was carried out in R, and the interactive visualizations were produced with the R Package Shiny.

## 3. Results

**Figure 4:**
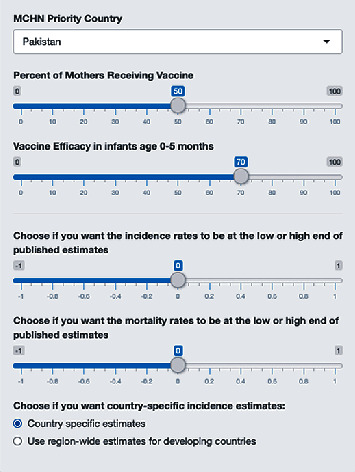
Control panel for interactive tool. The control panel of the web-based interactive tool that is used to assess the efficacy of candidate maternal RSV vaccination implementation at reducing infant disease incidence and mortality in developing countries is depicted. The user selects a country from the USAID MCHN priority countries list, the percentage of expectant mothers to receive the vaccine, and maternal vaccine effectiveness at preventing RSV in infants age 0-5 months. The user then chooses if they want incidence and mortality rates to be calculated using the low or high end of published estimates. Finally, the user selects if they prefer data to be displayed by country-specific or region-wise estimates.

We have developed a web-based interactive tool that can be used to assess the efficacy of candidate maternal RSV vaccination implementation at reducing infant disease incidence and mortality in developing countries. Using the control panel (Figure 4), the user selects a country of choice from the list of USAID's MCHN priority countries, the percentage of pregnant mothers expected to receive the vaccine, and maternal vaccine effectiveness at preventing RSV in infants age 0-5 months. Additionally, the user can decide their confidence in the calculations by using the web controls to adjust toward the low or high end of estimated values. Data can also be displayed using country-specific or region wide estimates. The resultant values are directly compared to the incidence and mortality estimates with no vaccine intervention.

**Figure 5:**
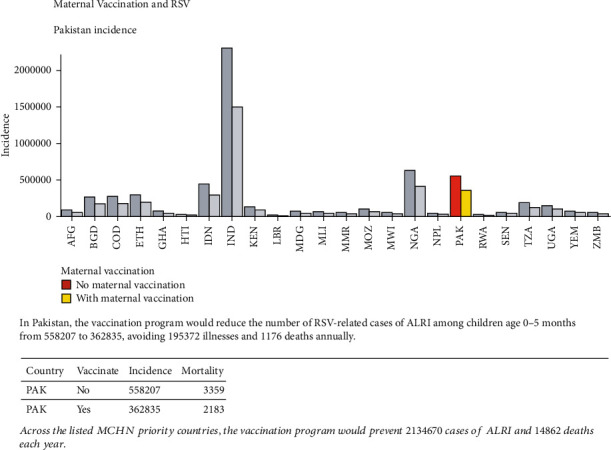
Algorithm visualization. An example of the visualization produced from the online tool. If the user is interested in implementing a candidate maternal RSV vaccine in Pakistan, they would select this country from the dropdown menu. If the distribution to pregnant mothers was estimated at 50% and the efficacy was estimated at 70%, over 195,000 cases of RSV and over 1,100 RSV-ALRI mortalities could have been avoided in Pakistan alone. The algorithm also reports that if this program were implemented in every USAID MCHN priority country, over 2 million cases of RSV and nearly 15,000 infant mortalities could be avoided annually.

An example of the visualization of the interactive tool is shown in Figure 5. If the user is interested in implementing a candidate maternal RSV vaccine in Pakistan, they would first select this country from the dropdown menu. They could then set a vaccine distribution and efficacy. If the distribution to pregnant mothers was estimated at 50% and the efficacy was estimated at 70%, over 195,000 cases of RSV and over 1,100 RSV-ALRI mortalities could have been avoided in Pakistan alone. The algorithm also reports that if this program was implemented in every USAID MCHN priority country, over 2 million cases of RSV and nearly 15,000 infant mortalities could be avoided annually.

The absolute reduction in incidence and infant mortality are most dramatic in countries with higher birth rates such as India, Nigeria, and Pakistan.

This tool is unique in that the user can determine the variables to best fit their purpose. It can give perspective and account for a highly effective vaccine that has decreased distribution due to cost or need for refrigeration or a vaccine with lower efficacy but a greater ability to be distributed.

## 4. Discussion

The treatment and prevention of RSV-related ALRI is multifaceted; maternal vaccination provides only one approach for reducing disease burden and mortality. Extensive effort has gone into developing childhood vaccines and pharmaceutical treatments. Conducting experimental RSV treatments on children and infants has proved difficult due to the sensitivity of the population and disease acuteness [10]. We chose to investigate the effects of maternal vaccination due to its well-established record of being an effective system for infant disease and mortality prevention [20]. Tetanus, Pertussis, and Influenza are examples of routine prenatal vaccines that could model the efficacy of maternal RSV vaccine implementation [21]. The distribution of existing vaccines can provide predictive power in determining the distribution of candidate vaccines. Furthermore, similar algorithms could be generated to visualize the effects of these existing vaccines on reducing disease burden in developing countries.

Anticipation of maternal RSV vaccine availability has led to a number of mathematical tools to estimate effectiveness of vaccine implementation. A model created by Hogan et al., based on data from Western Australia, reports that an RSV vaccine with a distribution of 50% and an efficacy of 80% could reduce hospital admissions by 26% for infants <3 months of age and 40% for infants age 3-5 months [20]. Data from Kenya estimated that maternal vaccination could reduce infant RSV incidence rates by up to 31.5% [22]. A predictive model created by Scheltema et al., using data from the United Kingdom and the Netherlands, estimated reductions in NICU admissions by 62% and RSV-related in-hospital infant deaths by up to 48%, given administration of variably effective maternal RSV vaccination at 30 weeks gestation [23]. These studies share the common expectation with our model that efficacy of passive immunity decreases dramatically as infant age increases and transient maternal antibodies decay.

Rainisch et al. also utilized the Shi et al. [1] data set to generate a model to assess RSV infant immunity strategies in the United States [24]. Three different potential immunization strategies were compared by effectiveness of reducing outcomes: monthly palivizumab therapy for high-risk infants, infant indicated one-time antibody therapy for all infants, and maternal vaccination for all plus palivizumab for high-risk infants. Based on their calculations, an infant indicated antibody treatment would prevent the largest number of out-patient visits, emergency department visits, and hospitalizations in the United States [24]. However, the study did not include a strategy solely dedicated to maternal vaccination, as is the focus of our model. Additionally, the impact of all immunization strategies on infant mortality was limited. Our model found greater impacts on mortality reduction with maternal vaccination likely due to the higher incidence and mortality rates in developing countries.

Other infectious diseases that cause significant infant mortality, such as Group B Strep, could be assessed in a similar manner. Group B Strep (GBS) is detected in the vaginal flora of 10-40% of reproductive age women [25]. Fetal and infant infection with GBS can have severe outcomes including preterm birth, neurological consequences, or death. While most developed countries test for GBS intrapartum and provide prophylactic intrapartum antibiotic treatment, this practice is not widely used in developing countries [25]. An estimated 90,000 infants who become infected with GBS die annually, and another 57,000 stillbirths can be associated with ascending GBS infection [17]. As with RSV, the burden of infant mortality and stillbirth is higher in developing countries [17]. The World Health Organization has recognized the development of a maternal GBS vaccine as a priority [26]. GlaxoSmithKline's GBS trivalent vaccine has shown favorable results with effective transplacental antibody transfer to infants for up to 3 months of age [15, 27]. Using GBS infection data from developing countries, our RSV algorithm could be used as a model for an adjustable tool in the assessment of candidate maternal GBS vaccines.

A major limitation of this project was the manipulation of data to fit the target population of infants age 0-5 months in USAID MCHN countries. Due to the subjectivity of the calculations and mathematical assumptions made, there is possibility of error in our estimates. For this reason, we have given the user opportunities to adjust the algorithm as necessary, including adjusting calculations to fit low or high ends of published estimates of disease incidence. In the future, if new data become available, we can make direct comparisons to our model and adjust accordingly to provide the most accurate and up-to-date estimates.

## 5. Conclusion

The adjustable algorithm provides a standardized analytical tool in assessing the effectiveness of candidate maternal RSV vaccination on reducing infant disease and mortality in developing countries. As vaccines continue through clinical trials and become widely available, this algorithm can provide foresight into vaccine application. Ultimately, it can be used to guide public health initiatives, research funding, and policy implementation concerning the impact of RSV on developing countries.

## Data Availability

The RSV incidence and mortality data supporting this algorithm generation are from previously reported studies and datasets, which have been cited. The processed data are available in the published works and supplementary files by Shi et al. [1]. The generated algorithm can be viewed and used at https://chrisleary.shinyapps.io/RSVVaccination/.
